# In-PREP: a new learning design framework and methodology applied to a relational care training intervention for healthcare assistants

**DOI:** 10.1186/s12913-020-05836-9

**Published:** 2020-11-04

**Authors:** Heather Wharrad, Sophie Sarre, Justine Schneider, Jill Maben, Clare Aldus, Elaine Argyle, Anthony Arthur

**Affiliations:** 1 School of Health Sciences, University of Nottingham, Nottingham, NG7 2HA UK; 2 School of Nursing, Midwifery and Palliative Care, King’s College, London, UK; 3 School of Law and Social Sciences, University of Nottingham, Nottingham, UK; 4 School of Health Sciences, University of Surrey, Guildford, UK; 5 School of Health Sciences, University of East Anglia, Norwich, UK; 6 School of Education, University of Nottingham, Nottingham, UK

**Keywords:** Intervention development, Continuing professional development, Older people, Training, Healthcare assistants, Co-design, Relational care, Educational theory, Pedagogical design

## Abstract

**Background:**

‘Older People’s Shoes’ is a training intervention designed for healthcare assistants (HCAs) to improve the relational care of older people in hospital. The intervention formed part of a broader evaluation, in this paper we describe its development from a learning design and methodological perspective.

**Methods:**

Learning theory and an instructional design model were key components of the In-PREP (Input, Process, Review and Evaluation, Product) development methodology used in the design of the ‘Older People’s Shoes’ training intervention to improve the delivery of relational care by front-line hospital staff. An expert panel, current evidence, and pedagogical theory were used to co-design a training programme tailored to a challenging work environment and taking account of trainees’ diverse educational experience. Peer review and process evaluation were built into the development model.

**Results:**

In-PREP provided a methodological scaffold for producing evidence-based, peer-reviewed, co-designed training. The product, ‘Older People’s Shoes’, involved a one-day Train the Trainers event, followed by delivery of a two-day, face-to-face training programme by the trainers, with accompanying handbooks underpinned by a range of digital resources. Evaluation found the approach met learner needs, was applicable in practice and won approval from trainers.

**Discussion:**

In-PREP enables high quality learning content, alignment with learner needs and a product that is relevant, practical and straightforward to implement.

## Background

The use of educational theory and instructional (pedagogical) design frameworks to guide the development of training interventions is important to ensure that the training meets the needs of the learners and to guide course designers. The UK’s Medical Research Council (MRC) guidance for the development of complex interventions [1], and the template for intervention description and replication (TIDieR) checklist and guide [2] have highlighted the need to pay careful attention to the design and reporting of all the elements of an intervention to permit replication and assessment of its efficacy. As part of a commissioned study titled ‘Can Health-care Assistant Training improve the relational care of older people?’ (CHAT), we embarked on uncovering how to approach the design of our training intervention using a rigorous development methodology.

This paper therefore details the pedagogical framework underpinning our approach and how this informed the process of designing and developing the evidence-based training package called ‘Older People’s Shoes’. Full details of the subsequent evaluation are reported elsewhere [3]. Here we describe the theory and process behind the design, and identify transferable lessons for practice education and for research. The use of an explicit, evidence-based framework to guide the development of training interventions, and the lessons learned from its application are relevant to future education and training initiatives for the healthcare workforce. They may be particularly useful in planning the development of those practitioners whose roles are new or lie outside established nursing and allied professionals’ educational pathways.

This paper describes the theory and process behind the design and development of a training programme on relational care for healthcare assistants (HCAs). Although the specific role titles may vary, nearly all health systems globally involve a large healthcare support workforce, often with minimal training [4]. HCAs have been deployed widely in hospitals and long-term care settings to undertake direct patient care related to essential care needs and activities of daily living [5], freeing nurses to focus on clinical responsibilities. Healthcare assistants outnumber nurses in the UK by three to one, and the proportion of HCA time delivering direct and indirect patient care is approximately 60%, nearly twice that of registered nurses [6]. The frequent and personal nature of this workforce’s ministrations means that their emotional intelligence and their capacity to respond to people’s needs are major determinants of the quality of care. Increasingly, patient experience and outcomes are mediated by contact with HCAs and other unregistered personnel (porters, receptionists for example). The HCA front line is therefore a crucial element in care. The growth in the HCA workforce has not been matched by an expansion in the opportunities for education and training [7]. Hence there is a compelling need for high quality training for this group of healthcare support workers.

## Methods

In previous work, in which we developed training and education interventions for healthcare professionals and patients, we have reported on a development methodology comprising a number of key stages leading to high quality design and content resulting in increased knowledge, confidence and behaviour change in the recipients [8–10]. The original work [8] identified three key elements that were important:

(i) the need to involve a community of stakeholders in ‘unlocking content’ workshops, including representatives of the trainee community who would be receiving the training as it became clear that their voice was crucial in ensuring the final product was closely aligned to their needs;
(ii) an understanding of pedagogical principles such as granularity of the learning materials and active engagement of the trainees;
(iii) the need for iterative quality review steps and formative evaluation prior to launch of the training package [8].

In later studies, this process was adapted to include an additional stage where more evidence was gathered prior to the content development workshops to inform what material should be covered in the training. This could be a Delphi survey for example [9, 10]. There were two reasons for producing this new framework, In-PREP, for this study. We wanted to produce a more generalised framework that was flexible enough to be used in a variety of training/education contexts for example by having ‘Inputs’ as the first step it is possible to draw on a range of different evidence to inform the training. Secondly, the previous frameworks were set up for digital training, whereas Older People’s Shoes was predominantly classroom based (with PowerPoint and supporting website) so the technical development phase of the process in the previous examples were not relevant to this particular training intervention.

**Fig. 1:**
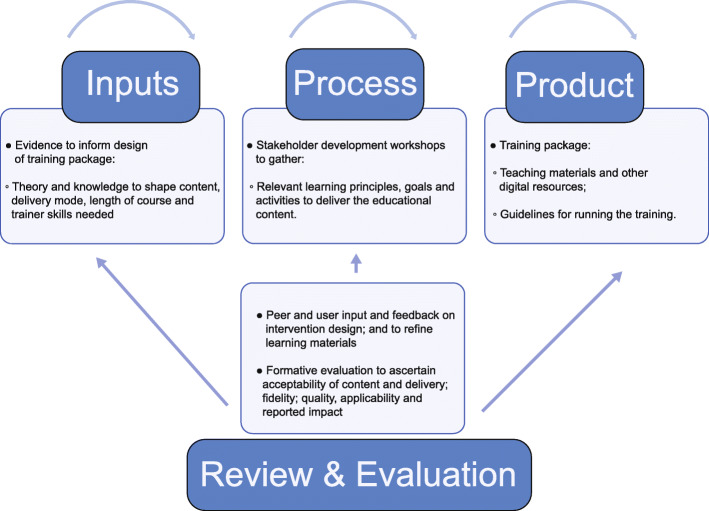
In-PREP consists of four activities outlined here. Inputs refer to the research-based evidence collected to identify the key decisions underpinning thetraining. Process refers to the co-design approach to create the content of the training guided by learning theory and pedagogical design. Review andEvaluation is an iterative cycle of checking and testing to ensure the fidelity, quality, acceptability of the training programme. Thr Product is the completedtraining package including teaching materials, digital resources and trainer guidelines

In-PREP is a participatory framework involving co-design, iterative review, evaluation and feedback in a virtuous cycle of innovation and improvement. The In-PREP process is shown in Fig. 1.

In-PREP consists of four activities outlined below, which include iterative review and evaluation throughout.

Inputs – a research-based approach to the following questions: What are the training needs of learners? What should the training consist of in terms of content and underlying principles and values, and how should the training be structured and delivered?

Process – participatory stakeholder co-design events to decide on the content and delivery of the training drawing on the evidence collected during the Input stage and guided by learning theory and pedagogical design.

Review and Evaluation – an iterative cycle of review and evaluation, including an evaluation of fidelity, quality, acceptability; and reported impact.

Product – training package produced which included teaching materials, digital resources and guidelines.

The following sections provide details of the development of the training intervention (‘Older People’s Shoes’) for each stage of the In-PREP framework to illustrate the process through which we identified and implemented the learning activities and delivery approach most suitable for HCA learners working in older people’s hospital wards.

### Inputs (**In-**PREP)

Four main sources informed the design: existing literature, current practice in HCA training nationally, the perspectives of healthcare stakeholders in three diverse regions of England, retailers with broad knowledge of customer care, and experts in the field. Detailed findings from our data collection are reported in more detail elsewhere [3, 11], but here we outline how the different inputs shaped our training product.

1. Literature review and qualitative synthesis study: An existing review and qualitative synthesis of published literature [12] identified three elements of relational care that contributed to positive experiences for recipients: the ‘connection’ between the carer and the patient and their relatives; maintaining their identity as a person; and being included in decisions about care. Existing initiatives and tools reported in the literature to improve relational care by healthcare staff were reviewed and helped to shape the content of the Older People’s Shoes training [13–20]. These included simulation training and life story tools.
2. Focus groups, stakeholder interviews and a national survey: We held three focus groups with a mix of older people with recent direct and indirect experience of hospital inpatient care – one in each of the three regions (total participants *n* = 29). We also carried out one-to-one interviews with HCAs (*n* = 30) and other ward staff (*n* = 24) who worked alongside or managed the HCAs in three acute hospitals. These interviews covered: training needs; optimum style and format of training; barriers and promoters of training take-up. During the latter stages of the input stage we sought input on our emerging ideas for the intervention from a subset of HCA interviewees. These interviews included a handout outlining the intervention and asking for feedback. The handout briefly outlined the intervention’s title, purpose, topics covered, timing, structure, delivery methods and underlying values.

A national telephone survey of 113 NHS acute hospital Trusts gave us a broader picture of HCA training. We used these findings to create meaningful learning activities that would allow HCA trainees to reflect on their current and future practice. The modes of delivery preferred by the HCAs and their trainers were practical and interactive based on real experiences, recognising and building on participants’ strengths; and enabling trainees to understand what it is like to be an older person in hospital. We also used the information to inform implementation of training. For instance, hospital staff suggested one-day blocks of training were most practicable to facilitate attendance; and preferred a blended approach, including practical exercises. Dependence on e-learning was not popular because of variable computer skills and limited access to computers in the workplace. There were also fears that the protected time needed to complete online training would not be available.

3. Learning from retailers: Four large commercial/retail organisations, selected for their alignment with entry-level roles training and for their work in valuing of older people, agreed to discuss with us their approach to improving customer care and shared their staff training content and delivery. Discussions with customer service trainers in these organisations covered a range of topics including learning theories and principles used to underpin their training. They told us that effective customer care training should convey: an understanding of the impact of good and bad practices in customer care; how to listen actively; why every interaction matters; why first impressions matter; the art of noticing things relevant to the person; and how to deal with challenging customers.
4 Consultation with national and international panel of experts: We consulted seven international experts about the training intervention development. They included researchers in the field, workforce directors, and healthcare learning advisors. Experts joined the development workshops with the research team in person or by video/audio link and were invited to provide evidence to inform our intervention and provide their critical views on our emerging thinking.

The evidence derived from these four sources shaped the underlying values and principles relating to effective delivery of the Older People’s Shoes training intervention, as illustrated in Table 1.

**Table 1:** Underpinning values and principles

Underlying values	
• Build on the assets that HCAs already bring to the provision of relational care	
• Team support is vital	
• Celebrate achievement	
• The importance of small actions	
• The power of communication	
Delivery principles	
• Protected time	
• Face-to-face learning	
• Online resources provided for reference	
• Layered curriculum approach [21] where the three units were introduced on day 1 and built on in day 2 the following week with a take home reflective exercise to do in-between	
Instructional Design	
• Clear take-home messages	
• Real life examples	
• Emphasis on learner interaction	
• Practical learning exercises	
• Encourage empathy through simulation	

### Process (In-**P**REP)

The design process was informed by learning theory and an instructional design model. These were brought to a series of training development events. We followed the MRC guidance, which includes cycles of checks and review in the development of complex interventions, along with a process evaluation of such interventions [1].

Braungart & Braungart provide a review of learning theories and their application to healthcare practice [22]. They state the importance of choosing the most appropriate theory and principles for a particular education experience. We used Carver’s [23] derivation of experiential learning theory [24] because it provided a conceptual framework which included principles relating to the training program design; the learning environment in which the training took place and in which the training would later be applied (hospital ward) and the ABC (personal agency, sense of belonging, and development of competence) of student experience. According to the ABC philosophy, the HCAs would draw from the training, the extent to which the locus of control was within themselves, see themselves as members with rights and responsibilities, and gain competence in applying what they learn. Four key elements of Carver’s programme design principles were particularly important in guiding all the inputs to the training content and design. These were the authenticity of the material, the active involvement of learners, validation of their respective experiences, and the importance of equipping participants to generalise their learning to new situations.

Instructional (pedagogical) design model: Within the overall theoretical framework afforded by Carver’s approach, we needed a more specific model to structure and link the elements of the training programme. Common pedagogical models offer a rationale for the teaching and learning process at the level of the individual learner or groups of learners. Such models help to ensure a consistency in pedagogical approach and provide a reference-point for resolving challenges raised by collective critique of the training programme under development. Gagné’s model [25] has frequently been used to guide the development of courses to teach procedural or practical skills [26–30]. It considers three important domains that impact on learning: how people feel, what they think and what they do, and so it is particularly suited to an experiential intervention on relational care. Therefore, when designing the training at the teaching session level, we developed the learning activities using Gagné’s learning design framework. In brief, for each learning activity within the units, this comprised setting objectives, offering trigger(s), presentation of information, practical or reflective activity around the information, and highlighting key messages.

Development co-design workshops: The training intervention Older People’s Shoes took shape over several iterations of development workshops. Development team members comprised: researchers members representing nursing, health services research, educational pedagogy, and social science; an HCA; and two Patient and Public Involvement (PPI) representatives. Members of the development team met together as needed over the intervention development period of the study. Joint decisions were made by the group to structure the intervention and develop content, based on the evidence, the chosen learning theory and pedagogical framework thereby refining the learning materials over several iterations.

Delivery of prototype intervention: A ‘train-the-trainer’ approach was adopted. Trainers were all registered nurses with training or project development responsibilities employed in each of the three hospitals where the training took place. This approach was supported by existing evidence that train-the-trainer programmes are effective in producing effective dissemination for learning in healthcare settings [31]. It had a number of pragmatic advantages including intervention sustainability beyond the life of the project, awareness of the local organisational context, and familiarity with the local workforce. The complete package for delivery of the training comprised a trainer guide and a trainee course book (Additional file 1).

### Review and evaluation (In-P**RE**P)

Throughout the input stage, we sought feedback on our developing ideas on the intervention design, and in the process stage we worked with stakeholders to design the intervention and refine the learning materials. The result, our ‘product’ was a novel training package, called Older People’s Shoes. During and after implementation of our pilot we systematically monitored and evaluated the content and its implementation using a number of methods [3].

For all train-the-trainer sessions, we used a structured observation template to assess engagement and learning on relational care; and to identify areas of improvement in the content and delivery of the training. These observations were discussed with the trainers after each session and their feedback sought. In this way researchers and trainers worked together to make small improvements to subsequent sessions; and ideas for any more substantial changes were captured. Observations were also used to evaluate fidelity of delivery (across sites and between trainers).

Evaluation forms for all HCA learners: At the end of each training day, time was built in for all HCA learners to complete an anonymous evaluation form, which used closed and open-ended questions to ask for their views on the training as a whole and the different activities within it, the resources, perceived impacts and any expected changes in their practice. Post intervention interviews with all six trainers explored their views on the content of the training, the usability of training resources and the support they received to deliver the training. We also asked for suggestions for improvement, and their views on the applicability of the training and the perceived impact of the intervention for HCAs. Post intervention interviews with 12 HCA learners were undertaken and included their experience of the intervention (including any suggestions for improvement) and any impacts on their practice. The results from the evaluations are shown in the Results section in Table 2.

**Table 2:** Insights from review and evaluation

Self-reported impacts:	
• Self-reported impacts were drawn from *n* = 81 evaluation form responses and 12 follow-up interviews with attendees. A few HCAs commented that they thought the training would be most appropriate for new HCAs, but all learning new things.	
• On evaluation forms from day 1 and day 2, 85 and 92% of HCAs, respectively, reported that as a result of the training they planned to make changes to the way they related to older people.	
• During follow-up interviews most interviewees were able to give us specific examples of changes they had made in their care practices since attending the training.	
• HCAs interviewees also reported changes in attitudes. They spoke about realising ‘how important the person underneath is’; the value of a good welcome; how much older people had lived through; and the effort and concentration many older people needed to do everyday tasks.	
• Six of the interviewees reported changes in the way they felt about their role: how important it was; what a difference they could make to people; how the recognition the training gave them made them feel more valued.	
The delivery of Older People’s Shoes:	
• Using trust-based trainers to deliver the training gave credibility, and their use of examples from their own experience on the ward and knowledge of the organizational context was well received.	
• Giving HCAs time off the ward to reflect on their work, discuss difficulties and share good practice with peers was regarded as a positive experience; and the assets-based approach made HCAs feel valued.	
• Trainers and HCA learner interviewees reported that the 2-day ‘layered curriculum’ structure worked well. One week’s gap between the days allowed for reflection and practice, which helped deeper learning.	
• Evaluation forms indicated that HCAs enjoyed the variety of learning approaches. They commented positively on being able to participate in discussions, the videos, the practical elements, the interactive approach and learning from others’ experiences.	
• During training HCAs commented on the professional quality of the course book and appeared to read this as a signifier of the value that was being placed on them.	
• Our observations within and across training centres were invaluable for monitoring the fidelity of implementation. We identified 21 deviations from fidelity. Most related to practical issues such as time-keeping and use of IT and other resources; or to general delivery and deviation from the trainer manual.	
• Not all deviations were negative. The trainers had a wealth of experience, and some of their innovations were evaluated as enhancing the training intervention as designed. The monitoring of fidelity meant we were able to take mitigating actions during implementation (as part of our continuous review and evaluation) and propose further changes for any future re-design.	

Patient and HCA outcomes were formally tested as part of a feasibility cluster-randomised controlled trial reported elsewhere [3]

### Product (In-PRE**P**) – the training package

In the following text, aspects that are consistent with Carver’s principles [23] are illustrated in **bold text** and those that correspond to steps in Gagnés framework [25] are indicated by underlined text.

#### Unit 1 understanding what it is like to be old, or getting into older People’s shoes

**HCAs were asked to remember** their first day on the ward as a trigger to explore the importance of the HCA role in making patients and families feel welcome in an unfamiliar ward environment. This drew on their **prior experience** and **assured them of its value** to the current session, while **engaging them actively** in the course. Patients’ experiences were **brought to life** by presenting talking heads short film clips in which **real** older patients talked about their experiences (both good and bad) of hospital care. The trainer fostered **interaction through discussion** around these clips and encouraged **sharing of experiences** sparked off by the talking heads. A reflective discussion on empathy, using an animation to show the difference between empathy and sympathy, followed by a group discussion on examples of empathy. Additional learning material consisted of ‘Today is Monday’ [32] a ‘fly on the wall’ film shot with **real care staff and patients** on a ward for older people with dementia. Learners had the opportunity to use age simulation suits, which simulated restrictions in movement, vision, hearing and touch providing **experiential learning** [24].

#### Unit 2 seeing the person behind the patient, or getting to know older people

This unit challenged HCA learners to think about how hospitalisation can strip away much of a person’s identity; and how stereotypical notions of ageing may lead care staff to make false or limiting assumptions about older people. It offered examples of ways to ‘discover the person behind the patient’ – such as through **rich life stories** using fictional characters aged 80, 90 and 100. A visual storytelling activity was supplemented with an activity based on still images of centenarians by photographer David Bailey. A discussion guided by **real quotes** from patients, HCAs and other ward staff, focused on the challenges and benefits of HCAs getting to know each of their patients. **Quotations from qualitative interviews with HCAs** [11], gathered by the research team, were used as triggers for learning. Two film clips were used to stimulate discussion about how knowledge about an older person’s history can affect how they are cared for and give important insights into their behaviour. The final part of this unit focused on the ingredients needed to build a relationship and, given the realities of a busy ward environment, the importance of practising relational care within everyday tasks to build stronger relationships with older people without putting further demands on HCAs’ time.

#### Unit 3 learning from customer care

During this unit, trainers **asked HCAs** to consider how some aspects of customer care provided in non-health settings may apply to their work in the ward. As a trigger, the group considered a situation (often outside work) when they had experienced good or bad customer care, what feelings these evoked, and what made these experiences different and memorable. At this point, the unit draws on some of the learning points gleaned from retail partners such as ‘active listening’, ‘how every interaction matters’ and the ‘art of noticing’. These concepts were reinforced in a training film originally used by a travel agency to illustrate how dramatically an experience of a service can be enhanced positively or affected negatively by the attitude, interest and behaviour of frontline staff members. An interactive discussion on being on the front line of patient care explored the demands of a healthcare environment, which challenged customer care principles, and how HCAs often have to manage difficult situations such as dealing with angry patients and visitors. This activity encouraged **peer-to-peer learning** by facilitating **reflection** and **discussion about strategies HCAs themselves** have found worked for them as well as providing tips for building on these ideas.

#### Structure and mode of delivery

Older People’s Shoes was delivered at three acute hospitals in workshop settings over 2 days made up of three units. Each unit explores one key aspect of relational care and is covered on both days, so that learning on the first day may be consolidated and built upon on the second day, approximately 1 week later (‘layered curriculum model’ [21]).

The training took an ‘assets-based’ approach by building on strengths and experiences of learners and emphasised the importance of affirming the HCA role in patient care, and celebrating successes. While acknowledging the importance of the team-based nature of care, we consciously avoided subsuming HCAs learning needs with that of the wider team. The training acknowledged the emotional impact on HCAs of delivering relational care and stressed the importance of self-care. HCAs were provided with details of their employers’ welfare support provision for any issues that may arise as a result of the training intervention or subsequently.

## Results

Table 2 reports findings from the evaluation component of the process relating to both what learners felt they had gained from undertaking the training, and what we learnt about the way the training was delivered.

## Discussion

Hoddinott [33] discussed the need to ‘open the black box of intervention development’ and the importance of methodological rigour during this phase of a complex intervention study. In this paper we have opened the black box to provide a detailed account of the process of developing Older People’s Shoes training for a complex intervention study drawing out transferable lessons for practice education emphasising the need to develop theoretically and evidence-based training for new roles and the expanding and diversifying workforce. It has applied a new training development methodology (In-PREP) to create a training intervention on relational care for HCAs. The key stages of In-PREP - Inputs, Process, Review, Evaluation and Product - led to a robust, evidence-based training product that was strongly aligned to learners’ training needs. It drew on multiple sources of research and expert evidence and a participatory approach to co-design and quality review. By using a rigorous development framework, we were able to produce a high quality, intensive training package which was favourably evaluated by trainers and HCA trainees with a wide range of expertise and prior training. This is important given the investment on the part of the employers in releasing HCAs for (in this case) 2 days of training.

We have shown here how a complex process drew on a wide range of evidence sources and applied sound pedagogical design to develop Older People’s Shoes. The need for transparency and detail in reporting intervention design has been noted [34, 35]. The training intervention was subsequently further tested as part of a feasibility cluster-randomised trial [3] in 12 hospital wards that tested the suitability of both HCA outcomes and patient outcomes. We have illustrated a promising use of structured development to meet the learning needs of HCAs concerning relational care in hospital. A realist synthesis approach which identified eight explanations as to what elements of development interventions work for the older person’s support workforce [36] including multiple stakeholder co-design and taking a planned approach that draws on theory.

The In-PREP approach is likely to have wider applications to other learners and other settings where a growing body of evidence underpins best practice and where groups of professionals must constantly acquire new knowledge and adapt their activities to take account of innovation and development.

## Conclusions

Application of the In-PREP model will ensure that training interventions like Older People’s Shoes are consistently designed to a high standard using a co-design approach.

The key principles of IN-PREP are: ensuring rigour during important steps such as identifying appropriate evidence as inputs to inform the training content and delivery, and the co-design approach during the process of development involving multiple stakeholders and quality review stages. The role of educational theory and pedagogical design ensured that training such as Older People’s Shoes not only addresses ‘what’ HCAs learn but optimises ‘how’ they learn within the constraints of their work situations and work-life balance. Evaluation during and shortly after the training showed that HCAs enjoyed and learned from the training. In-PREP provided an important methodological scaffold to the development of a practice-based experiential training intervention which has wide application. In-PREP guided processes shows great promise for the design of other training interventions for this and similar workforces.

## Supplementary Information

**Additional file 1.** Front cover and example pages from the ‘Older People’s Shoes’ training intervention for relational care of older people by Healthcare Assistants.

## Data Availability

N/A

## Abbreviations

OPS: Older People’s Shoes
CHAT: Can Healthcare Assistant Training improve the relational care of older people? (the short title of the study for which the OPS training was developed)
In-PREP: Inputs, Process, Review, Product development methodology framework
TIDieR: Template for intervention description and replication
