# Supplementary material for: In-PREP: a new learning design framework and methodology applied to a relational care training intervention for healthcare assistants
Source: BMC Health Serv Res. 2020 Nov 4;20:1010. doi: 10.1186/s12913-020-05836-9 (PMC7643258; doi:10.1186/s12913-020-05836-9)

Additional file 1 Front cover and example pages from the ‘Older People’s Shoes’ training intervention for relational care of older people by Healthcare Assistants


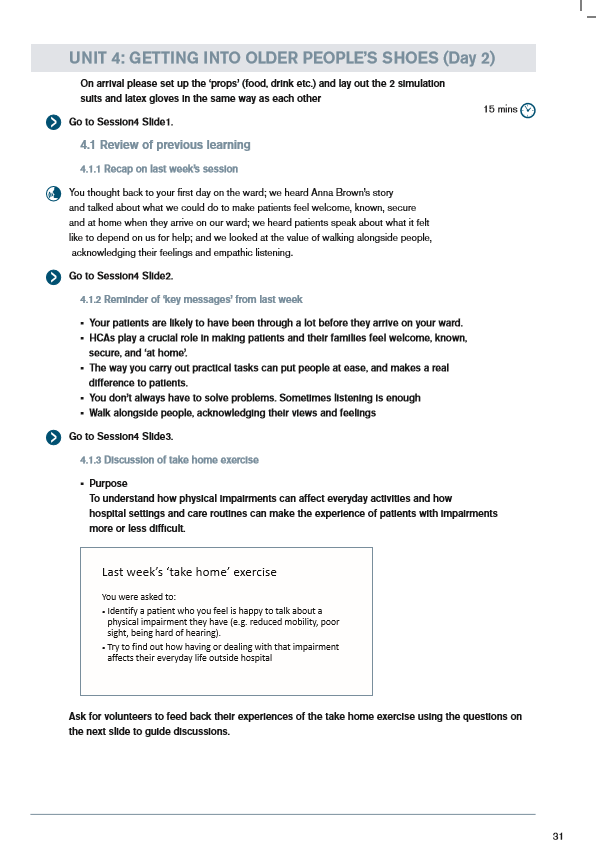

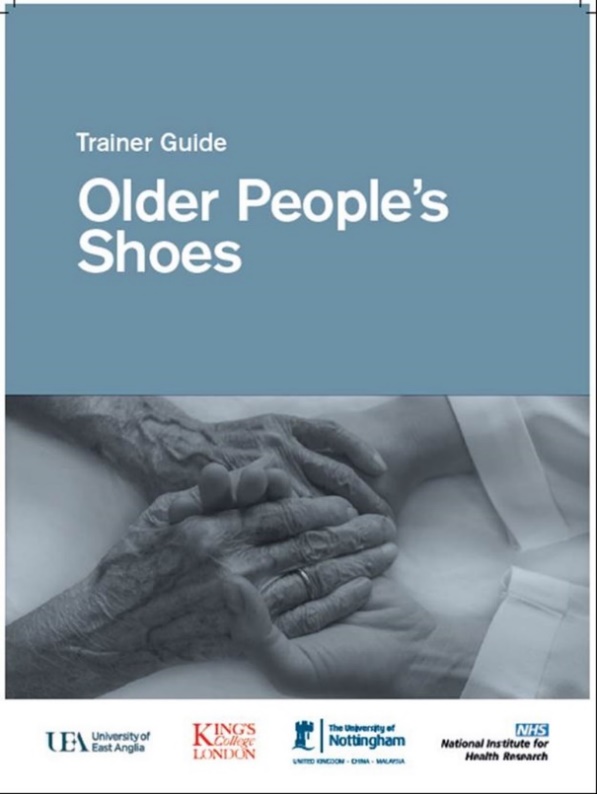


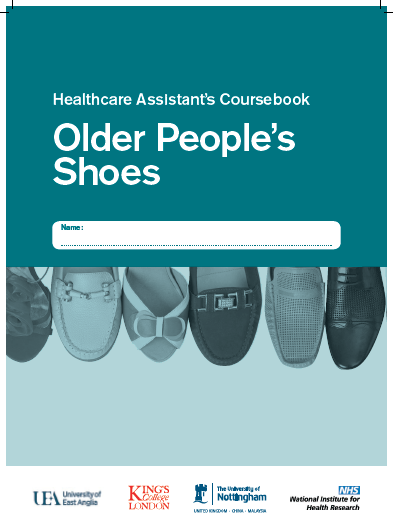


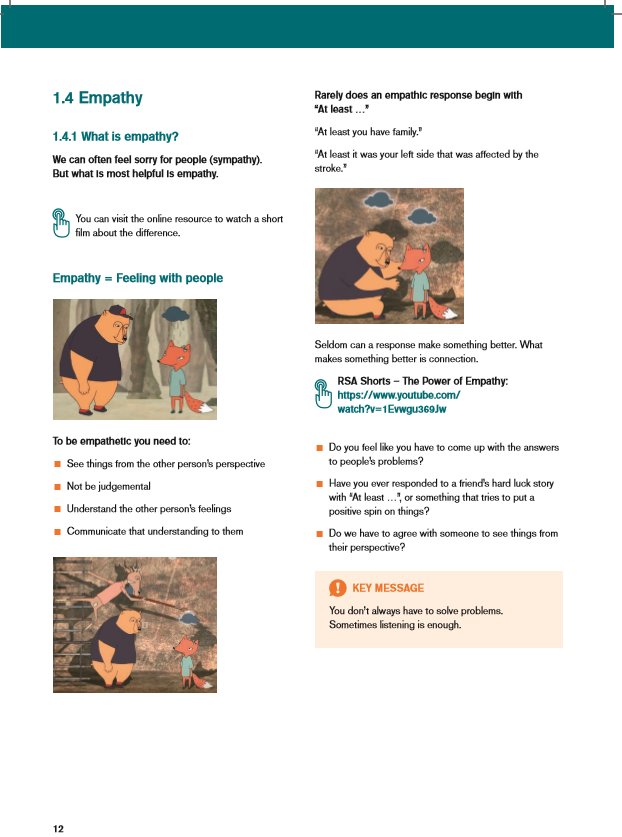

Supplement: Supplementary file 1 — Additional file 1. Front cover and example pages from the ‘Older People’s Shoes’ training intervention for relational care of older people by Healthcare Assistants. [file 12913_2020_5836_MOESM1_ESM.docx]
